# Complete Genome Sequence of Salmonella enterica Siphophage Shelanagig

**DOI:** 10.1128/MRA.01033-19

**Published:** 2019-09-26

**Authors:** Kathryn Broussard, Yicheng Xie, Heather Newkirk, Mei Liu, Jason J. Gill, Jolene Ramsey

**Affiliations:** a Center for Phage Technology, Texas A&M University, College Station, Texas, USA; Loyola University Chicago

## Abstract

Salmonella enterica is a Gram-negative human pathogen widely known to cause food poisoning. Here, the genome of S. enterica phage Shelanagig is described. Its 42,541-bp genome codes for 68 proteins, for which 33 were assigned a predicted function. Shelanagig shares high similarity at the protein level with other *Salmonella* phages.

## ANNOUNCEMENT

The Gram-negative bacterium Salmonella enterica is a bacterial human pathogen, causing illnesses such as gastroenteritis and typhoid fever (1). Outbreaks of S. enterica typically lead to many deaths, have high monetary costs, and spread via bacterial contamination of food products such as chicken or leafy greens. Phages that target this pathogen may be used to decrease the prevalence of S. enterica outbreaks through decontamination of food or food production facilities (2). Here, we report the genome sequence of phage Shelanagig, which infects S. enterica.

Phage Shelanagig was isolated from cattle holding pen soil samples collected in Michigan after processing as described by Xie et al. (3). The host, S. enterica serovar Enteritidis, was cultured on tryptic soy broth or agar (Difco) at 37°C with aeration. Phage were propagated using the soft agar overlay method (4). Phage samples were stained with 2% (wt/vol) uranyl acetate and viewed using transmission electron microscopy at the Texas A&M Microscopy and Imaging Center to ascertain morphology (5). The Shelanagig genome was purified by the shotgun library preparation protocol modification of the Promega Wizard DNA clean-up system (6). Illumina sequencing libraries were prepared with their TruSeq Nano low-throughput kit. The sequencing occurred by v2 500-cycle chemistry on an Illumina MiSeq platform with paired-end 250-bp reads. The 162,861 total reads in the index containing the phage were controlled for quality using FastQC (http://www.bioinformatics.babraham.ac.uk/projects/fastqc/). Based on that, the reads were trimmed with the FASTX-Toolkit v0.0.14 (http://hannonlab.cshl.edu/fastx_toolkit/). Assembly using SPAdes v3.5.0 yielded a contig with 74-fold coverage (7). The contig was fully closed by PCR (forward primer, 5′-GCTCAAGACAGTGAGCAGTAA-3′, and reverse primer, 5′-TTTACAGCCCATCTGTCGTG-3′) and Sanger sequencing. Genes were predicted with Glimmer v3.0 and MetaGeneAnnotator v1.0 (8, 9). tRNA coding was probed with ARAGORN v2.36 (10). The presence of Rho-independent terminators was predicted with TransTermHP v2.09 (11). Gene functions were then predicted using domain searching with InterProScan v5.22-61 and comparison via BLAST v2.2.31 to the NCBI nonredundant, UniProtKB Swiss-Prot, and TrEMBL databases using a 0.001 cutoff for the maximum expectation value (12–14). As needed, TMHMM v2.0 results were also inspected (15). Full-length nucleotide sequence similarity was calculated using progressiveMauve v2.4.0 (16). All annotation tools are hosted in the Galaxy and Web Apollo instances at the Center for Phage Technology (https://cpt.tamu.edu/galaxy-pub) (17, 18). Unless otherwise stated, all tools were executed using default parameters.

Shelanagig is a siphophage with a genome of 42,541 bp and 49.8% G+C content. It has 68 protein-coding genes on both strands, 33 of which have predicted functions, and the coding density is 94.1%. The phage contains no tRNA genes. The program PhageTerm predicts a headful packaging mechanism (19). While at the nucleotide level, Shelanagig has the highest identity to *Salmonella* phages ST3 (GenBank accession number MF001364) and ST1 (GenBank accession number MF001366), at 84.92% and 84.90% similarity, respectively, Shelanagig shares 59 proteins with *Salmonella* phages SETP7 (GenBank accession number KF562865), wksl3 (GenBank accession number JX202565), and BPS11Q3 (GenBank accession number KX405002). Interestingly, Shelanagig contains the slippery sequence needed to produce a frameshifted version of the tail assembly chaperone, as characterized in *Escherichia* phage lambda G and GT (20).

### Data availability.

The genome sequence and associated data for phage Shelanagig were deposited under GenBank accession number MK931446, BioProject accession number PRJNA222858, SRA accession number SRR8869227, and BioSample accession number SAMN11360386.
